# Development of Immunocapture-LC/MS Assay for Simultaneous ADA Isotyping and Semiquantitation

**DOI:** 10.1155/2016/7682472

**Published:** 2016-01-28

**Authors:** Lin-Zhi Chen, David Roos, Elsy Philip

**Affiliations:** Boehringer Ingelheim Pharmaceuticals, Inc., Ridgefield, CT 06877, USA

## Abstract

Therapeutic proteins and peptides have potential to elicit immune responses resulting in anti-drug antibodies that can pose problems for both patient safety and product efficacy. During drug development immunogenicity is usually examined by risk-based approach along with specific strategies for developing “fit-for-purpose” bioanalytical approaches. Enzyme-linked immunosorbent assays and electrochemiluminescence immunoassays are the most widely used platform for ADA detection due to their high sensitivity and throughput. During the past decade, LC/MS has emerged as a promising technology for quantitation of biotherapeutics and protein biomarkers in biological matrices, mainly owing to its high specificity, selectivity, multiplexing, and wide dynamic range. In fully taking these advantages, we describe here an immunocapture-LC/MS methodology for simultaneous isotyping and semiquantitation of ADA in human plasma. Briefly, ADA and/or drug-ADA complex is captured by biotinylated drug or anti-drug Ab, immobilized on streptavidin magnetic beads, and separated from human plasma by a magnet. ADA is then released from the beads and subjected to trypsin digestion followed by LC/MS detection of specific universal peptides for each ADA isotype. The LC/MS data are analyzed using cut-point and calibration curve. The proof-of-concept of this methodology is demonstrated by detecting preexisting ADA in human plasma.

## 1. Introduction

Therapeutic proteins and peptides have potential to elicit immune responses [1, 2], resulting in anti-drug antibodies (ADAs) that can pose problems for both patient safety and product efficacy. Clinical consequences can range from relatively mild to serious adverse events [3–5], such as anaphylaxis, cytokine release syndrome, and cross-reactive neutralization of endogenous proteins mediating critical functions. ADA can affect drug efficacy and biodistribution and drug clearance, and complicate the interpretations of toxicity and pharmacokinetic (PK) and pharmacodynamic (PD) data [6–8]. During drug development immunogenicity is examined by risk-based approach along with specific strategies for developing “fit-for-purpose” bioanalytical approaches [9].

Characterization and analysis of ADA are a vital element of immunogenicity assessment. Enzyme-linked immunosorbent assays (ELISA) and electrochemiluminescence (ECL) immunoassays [10] are the most widely used platform for ADA detection due to their high sensitivity and throughput. Lower affinity ADA can be detected by surface plasmon resonance, biolayer interferometry, or other platforms [11]. Typically, detection of ADA is followed by assessments of the magnitude (titer) of the ADA response and the in vitro neutralizing ability of ADA, especially in late-stage clinical studies. Additional characterization of ADA such as immunoglobulin subclass or isotype determinations, domain-mapping, relative binding affinity, cross-reactivity with endogenous proteins, or complement activating ability of the ADA may be driven by product-specific, indication-specific, or risk assessment-based objectives [9, 12, 13].

Recommendations for ADA assay development, method validation, and testing strategies have been published by the Ligand-Binding Assay Bioanalytical Focus Group (LBABFG) of American Association of Pharmaceutical Scientists (AAPS) [10, 12, 14–16]. Additionally, scientific publications on risk-based approaches to immunogenicity assessments [9, 12, 17–19] and regulatory documents from the US Food and Drug Administration (FDA) and the European Medicine Agency (EMA) are also available [20–23]. Together, these documents provide ample guidance for the application of appropriate ADA detection methods in clinical studies.

Since the late 1990s, liquid chromatography coupled to mass spectrometry (LC/MS) has been a dominant tool for sensitive, accurate, and rapid analysis of small-molecule drugs, metabolites, and biomarkers [24]. In recent years, LC/MS has emerged as a promising platform for quantitation of biotherapeutics and protein biomarkers in biological matrices [25–27]. The vast majority of LC/MS-based protein quantifications are performed at peptide levels, mainly due to consideration of assay sensitivity [28]. A typical procedure for LC/MS-based quantification includes enzyme digestion and quantification of the target proteins based on selected signature peptides derived from the target [29, 30].

Recently, Furlong et al. developed a universal peptide method to quantitate human antibody Fc region-containing therapeutic protein candidates in nonclinical species [31]. Surrogate tryptic peptide VVSVLTVLHQDWLNGK for IgG1, IgG3, and IgG4 and VVSVLTVVHQDWLNGK for IgG2 were identified in the Fc region of human immunoglobulins (IgG), respectively. The method was shown to be capable of supporting bioanalysis of a diversity of human Fc region-containing therapeutic protein candidates in plasma samples of all commonly used animal species, thus eliminating the need to develop unique peptide methods for each individual therapeutic candidate. With a similar approach, Dongen et al. achieved a higher sensitivity of 4 ng/mL for a monoclonal Ab drug, infliximab, using a different universal peptide (SLSLSPGK) from the C-terminal of Fc [32]. Li et al. used a stable isotope labeled common mAb as internal standard for quantitation of therapeutic mAb in preclinical samples [33]. Not only was the common whole Ab internal standard able to correct for variations from the beginning of sample processing to ionization in the mass spectrometer but also it allowed rapid method development with flexible choice of a suitable surrogate peptide for new applications, such as different species or different mAb. Stable isotope labeled human monoclonal Ab incorporating [^13^C_6_,^15^N_4_]-arginine and [^13^C_6_,^15^N_2_]-lysine is now commercially available and can be used for the universal peptide methods.

LC/MS has also been reported to assess ADA in the presence of excess protein therapeutic in support of clinical programs addressing the safety and tolerability of human growth hormone analogues [34]. This methodology overcame drug tolerance issues, which are often associated with traditional ADA detection [35–37], by completely saturating available ADA binding sites with the addition of excess therapeutic. Drug-ADA complexes were then isolated using protein G immobilized on magnetic beads, followed by elution and digestion. Resultant peptide from the target therapeutic proteins was quantified by LC coupled with matrix-assisted laser desorption MS and the results were correlated to the binding capacity of total ADA.

In another application, LC/MS was used to evaluate neutralizing Ab (NAb) assay by simultaneously quantitating residual mAb-drug, endogenous IgG, and NAb-positive control in BEAD eluates [38]. In the study, the low levels of the residual drug and human IgG in the BEAD eluates indicate that the BEAD efficiently removed the high concentration drug and serum components from the serum samples. Meanwhile, the NAb-positive control recovery (~42%) in the BEAD provided an acceptable detection limit for the cell-based assay. This novel application of LC/MS to immunogenicity assay development demonstrates the advantages of LC/MS in selectivity and multiplexing, which provides direct and fast measurements of multiple components.

We describe here an immunocapture-LC/MS-based approach for simultaneous ADA isotyping and semiquantitation in human plasma. Biotinylated drug or anti-drug Ab is used to capture ADAs or drug-ADA complexes in plasma, respectively. The resulting ADA-drug or ADA-drug-Ab complexes are then immobilized on streptavidin magnetic beads and separated from matrix by a magnet. After washing, ADA is released from the beads and subjected to trypsin digestion followed by LC/MS detection of specific universal peptides for each ADA isotype.

## 2. Materials and Methods

Protein Z (containing no human Fc) was a proprietary experimental biotherapeutic of Boehringer Ingelheim Pharmaceuticals (Ridgefield, CT) and produced in-house. The mouse anti-Protein Z monoclonal Ab (mAb) was supplied in-house. Human IgG1, IgG2, IgG3, IgG4, and IgM as well as bovine serum albumin (BSA) were purchased from Sigma Aldrich (St. Louis, MO), human IgE was from MP Biomedicals (Solon, OH), human IgA1 was from Abcam (Cambridge, MA), and human IgA2 was from EMD Millipore (Billerica, MA). Internal standard peptides with stable labeled C-terminal [^13^C_6_,^15^N_4_]Arg or [^13^C_6_,^15^N_2_]Lys were synthesized at GenScript (Piscataway, NJ). Streptavidin magnetic beads (1 *μ*m dia.), TPCK trypsin, and EZ-Link Sulfo-NHS-LC Biotinylation kits were obtained from Thermo Scientific (Rockford, IL). RapiGest SF was purchased from Waters (Milford, MA). All other lab chemicals, reagents, and buffer solutions were obtained from Sigma Aldrich, Thermo Scientific or Invitrogen (Grand Island, NY). Human preexisting ADA (PEA) positive and negative plasma were obtained in-house.

### 2.1. Biotinylation

Biotinylation of Protein Z and the mouse anti-Protein Z mAb was performed using an EZ-Link Sulfo-NHS-LC Biotinylation kit. A 1 mg/mL solution of the drug or mAb was combined with a 10-fold molar excess of biotin and allowed to react at room temperature for 60 minutes. Excess biotin was removed using desalting columns provided in the kit. A HABA assay was used to determine the amount of biotin incorporation in the sample after desalting. Typical biotin incorporation was approximately 2 biotins per drug and 4–7 biotins per mouse mAb. The biotinylated drug and biotinylated mouse mAb solutions were diluted to 0.1 mg/mL in water and stored at −80°C prior to use.

### 2.2. Immunocapture with Biotinylated Drug

Streptavidin magnetic beads were prepared freshly for each assay batch. The magnetic beads (10 mg/mL) were transferred to a polypropylene tube and placed on a magnetic stand to remove supernatant and collect the beads. The beads were then washed with 10x volume of Tris buffered saline containing 0.1% Tween-20 (TBS-T) and resuspended in 2x volume of TBS-T to yield a final working bead concentration of 5 mg/mL.

An aliquot of 95 *μ*L of human plasma sample and 5 *μ*L of 5.85 M acetic acid were pipetted into a 96 deep-well polypropylene plate. The plate was incubated with gentle mixing for 1 hr at room temperature. After adding 75 *μ*L aliquot of 0.1 mg/mL biotinylated Protein Z and 40 *μ*L of Trizma base (1.5 M Tris, pH 10) to each sample, the plate was incubated at room temperature for 1.5 hr. A 40 *μ*L aliquot of freshly prepared 5 mg/mL magnetic beads and 475 *μ*L of TBS-T binding buffer were added to each sample and the plate was gently mixed for 1 hr at room temperature. The beads were then separated, washed three times with 300 *μ*L of TBS-T and once with 300 *μ*L of water, and eluted with 150 *μ*L of 0.1 M glycine (pH 2.0) on a Kingfisher Flex bead handler (Thermo Scientific, San Jose, CA). The eluent was immediately neutralized with 45 *μ*L of 1 M Tris-HCl (pH 8.0) followed by the addition of 10 *μ*L of 0.1% BSA.

### 2.3. Immunocapture with Mouse mAb

An aliquot of 144 *μ*L of human plasma sample and 6 *μ*L of 5 mg/mL Protein Z aqueous solution were pipetted into a 96-deep-well polypropylene plate. The plate was incubated at 37°C with gentle mixing for 1 hr and then stored at −80°C overnight. A 100 *μ*L aliquot of 0.1 mg/mL biotinylated mouse mAb was added to each sample and the plate was then incubated at room temperature for 2 hrs. A 100 *μ*L aliquot of freshly prepared 5 mg/mL magnetic beads and 475 *μ*L of TBS-T binding buffer were added to each sample and the plate was gently mixed for 1 hr at room temperature. The beads were separated, washed three times with 300 *μ*L of TBS-T and once with 300 *μ*L of water, and then eluted with 150 *μ*L of 0.1 M glycine (pH 2.0) on a Kingfisher Flex bead handler. The eluent was immediately neutralized with 45 *μ*L of 1 M Tris-HCl (pH 8.0) followed by addition of 10 *μ*L of 0.1% BSA.

### 2.4. Matrix Calibration Standards

Commercial stock solutions of the different immunoglobulins (Igs) ranged from 1 to 4.18 mg/mL. A series of 50–10,000 ng/mL spiking calibration standards were prepared in 0.1% BSA using the stock solutions and stored at −80°C prior to use. Pooled human blank plasma was processed using either immunocapture procedure. Matrix calibration standards were prepared by adding 10 *μ*L of the spiking calibration standards to the magnetic bead eluent of the pooled human blank plasma.

### 2.5. Trypsin Digestion

To the immunocapture eluent, matrix calibration standards, or neat Ig PBS solution, 5 *μ*L of 0.1% RapiGest in 100 mM ammonium bicarbonate was added and the plate was gently mixed for 5 minutes. Five *μ*L of a stable labeled internal standard solution of the universal peptides (0.2 *μ*g/mL) and 5 *μ*L of 50 mM TCEP in 100 mM ammonium bicarbonate were added and followed by incubation at room temperature for 20 min. After adding 5 *μ*L of 50 mM iodoacetamide in 100 mM ammonium bicarbonate, the plate was gently shaken for 20 min while protected from light. A 5 *μ*L aliquot of solution containing trypsin (0.2 mg/mL) and calcium chloride (0.2 M), prepared immediately before use, was added to each sample and the plate was incubated at 37°C overnight with gentle mixing. Digestion was quenched by adding 5 *μ*L of 20% TFA. The samples were mixed for 40 min at 37°C and then centrifuged at 4400 rpm for 10 min prior to LC/MS analysis.

### 2.6. LC/MS Analysis

Eksigent Ekspert MicroLC 200 coupled with AB Sciex 6500 triple quadrupole mass spectrometer (AB Sciex, Framingham, MA) was used. Chromatographic separation was performed using ACQUITY UPLC Peptide BEH C18 column (1 mm × 50 mm, 1.7 *μ*m, 300 Å) operated at 60°C. Mobile phases consisted of (A) 0.1% formic acid and (B) 0.1% formic acid in acetonitrile running at a flow rate of 60 *μ*L/min. For information-dependent acquisition (IDA), the LC gradient was 5% to 50% B over 18 minutes. For ADA isotyping and semiquantitation, the LC gradient was 12% to 17% B over 2.8 minutes and then to 47% B over 6.2 minutes.

The mass spectrometer was operated in in positive electrospray ionization mode. Key instrument parameters were as follows: +5000 V electrospray voltage, 65 nebulizer gas units, 30 axillary gas units, 375°C ion source temperature, 10 collision gas units, and unit resolution on both Q1 and Q3. For identifying unique peptides for each ADA isotype with IDA, up to 4 multiple-reaction-monitoring (MRM) pairs were used for screening followed by enhanced product ion scan. For ADA isotyping and semiquantitation, 13 MRM pairs of universal peptides were used along their respective internal standards.

## 3. Results and Discussion

Despite its many advantages and potential, the use of LC/MS for protein quantitation is not as straightforward as for small-molecules and oftentimes demands comprehensive method development. Plasma and serum are very complex matrices that contain several hundreds of thousands of proteins and protein isoforms in a wide concentration range [39]. Upon digestion these are all cleaved into multiple peptides, from which just one or a few have to be quantified. When no protein or peptide purification is employed, LC/MS sensitivity is significantly compromised due to matrix interference from the peptide-rich digest. For high sensitivity LC/MS applications, immunopurification is the most effective way to improve assay sensitivity and robustness. Immunopurification can be done either at the protein-level prior to digestion [40, 41] or at the peptide level after digestion [42]. Immunopurification of peptides requires anti-peptide Ab for each peptide of interest. Unlike proteins, small peptides are usually less or even not immunogenic, which presents significant challenges for anti-peptide Ab production. Moreover, ADA and/or drug-ADA complex has to be pulled down prior to digestion and peptide pull-down. In consideration of these factors, we employed protein-level immunopurification for sample preparation.

Antibodies are secreted by plasma cells and come in different isotypes with genetic variations or differences in the constant regions of the heavy and light chains. In humans, there are five heavy chain isotypes (*α*: IgA; *δ*: IgD; *γ*: IgG; *ε*: IgE; and *μ*: IgM) and two light chain isotypes (*κ* and *λ*). In addition, IgG has 4 subclasses (IgG1, IgG2, IgG3, and IgG4) and IgA has 2 subclasses (IgA1 and IgA2). Relative Ab abundance (% total Igs) in human varies significantly among isotypes/subclasses: IgG1 (65%), IgG2 (25%), IgG3 (5%), IgG4 (5%), IgA1, IgA2 (13% IgA1 + IgA2), IgE (<0.003%), IgM (8%), and IgD (<1%). Abs can present as soluble and/or membrane-bound on the surface of B cells and only the soluble Abs can be found in plasma or serum [43]. All isotypes can be found in normal serum. Among them, IgG1–IgG4 are the most abundant antibody isotypes found in the circulation and provide the majority of antibody-based immunity against invading pathogens [44]. Although IgE is the least abundant isotype, it plays an essential role in type I hypersensitivity and allergic conditions [45, 46], such as anaphylactic reactions to certain drugs. IgA is an antibody that plays a critical role in mucosal immunity and is found in small amounts in blood. IgD makes up about 1% of proteins in the plasma membranes of mature B-lymphocytes but only represents about 0.25% of Igs in serum and is thus excluded in the immunocapture-LC/MS assay.

### 3.1. Selection of Unique Peptide

ADA isotyping and semiquantitation were based on the surrogate peptides of ADAs instead of whole molecules, mainly in consideration of sensitivity [28–30]. The semiquantitative measurement relied on the existence of a stoichiometric (quantitative) relationship between ADA and its surrogate peptide. By far the most critical element of this approach was to identify proper peptide(s) that were unique to each antibody isotype/subclass. Three main factors were considered in selecting the unique peptides. First of all, the surrogate peptide(s) to each isotype/subclass must come from the constant region. Although ADAs of the same isotype come in many different forms in terms of amino acid sequence in their variable regions, they all have the same constant region. Secondly, the surrogate peptides should be unique to each ADA isotype/subclass and should not be formed in any other isotypes. Thirdly, the surrogate peptides should not contain the amino acids of ADA allotype polymorphic residues [47]. Peptides that met the three criteria represented a certain ADA isotype regardless of differences in ADA variable regions, thus allowing ADA isotyping and semiquantitation by LC/MS.

Among the Ig isotypes, only IgG1–IgG3 and IgA2 have different allotypes [47]. At present, 26 human allotypes are known [47, 48]: 6 for IgG1 (G1m1, G1m2, G1m3, G1m17, G1m27, and G1m28), 1 for IgG2 (G2m23), 13 for IgG3 (G3m5, G3m6, G3m10, G3m11, G3m13, G3m14, G3m15, G3m16, G3m21, G3m24, G3m26, G3m27, and G3m28), and 3 for IgA2 (A2m1, A2m2, and A2m3). In addition, 3 allotypes were found for *κ* light chain (Km1, Km2, and Km3) [48]. Except for G1m3 and G1m17 located on the CH1, all heavy chain allotypes are localized on the Fc region, either on CH2 or on CH3. IgG1 heavy gamma chain allotypes differ in amino acid (AA) residues at 214, 356, 358, and 431. The heavy chains of G2m23 allotype differ in AA residues 214 and 282. IgG3 has the most allotypes, and they differ at AA residues at 291, 292, 379, 384, 397, 409, 419, 422, 435, and 436. For IgA2, A2m1, A2m2, and A2m3 differ at AA residues at 115 and 124. The unique surrogate peptide(s) for each isotype/subclass should be present in all of its allotypes. Since many isotypes share the same light chains (*κ* and *λ*), light chains were excluded from the peptide selection.

Additional criteria that are commonly used in selecting a surrogate peptide include the following [49]: (a) avoid peptides containing methionine which are prone to oxidation; (b) avoid peptides containing asparagine-glycine or aspartic acid-glycine, which are prone to deamidation; (c) avoid peptides containing N-terminal glutamine or glutamic acid, which are prone to N-terminal cyclization; (d) avoid peptide sequences containing arginine-arginine (RR) and lysine-lysine (KK), which yield inconsistent tryptic digestion; (e) keep away from peptide sequences containing arginine-proline (RP) and lysine-proline (KP) which are difficult to break down by digestion; (f) keep peptide length between 5 and 15 amino acids to minimize the number of ionization charge states, achieve efficient MS/MS fragmentation and high sensitivity, and obtain desirable chromatographic retention; and (g) avoid peptide sequences containing glycosylation sites.

To identify candidates for unique peptides, 15 *μ*g/mL of individual Ig PBS buffer solution was digested and the resulting digest was assayed by LC/MS using IDA. The IDA setup consisted of MRM survey scan for the formation of tryptic peptides based on in silico prediction, followed by enhanced product ion scans to confirm peptide identity. Only peptides that met all the criteria set forth as discussed above were included in the survey scan. Peptide separation was achieved with a 20 min ultrahigh performance liquid chromatography (UPLC) gradient program. The top 1–3 most sensitive peptides for each ADA isotype/subclass from the neat solution digestion were selected as unique peptide candidates and carried on for further method development. Of these peptides, either doubly or triply charged parent ions were the most abundant. Upon collision activation in MS/MS, the multiple charged parent ions fragmented into single charged b-ions and y-ions and the most sensitive y-ions were selected.

**Figure 1:**
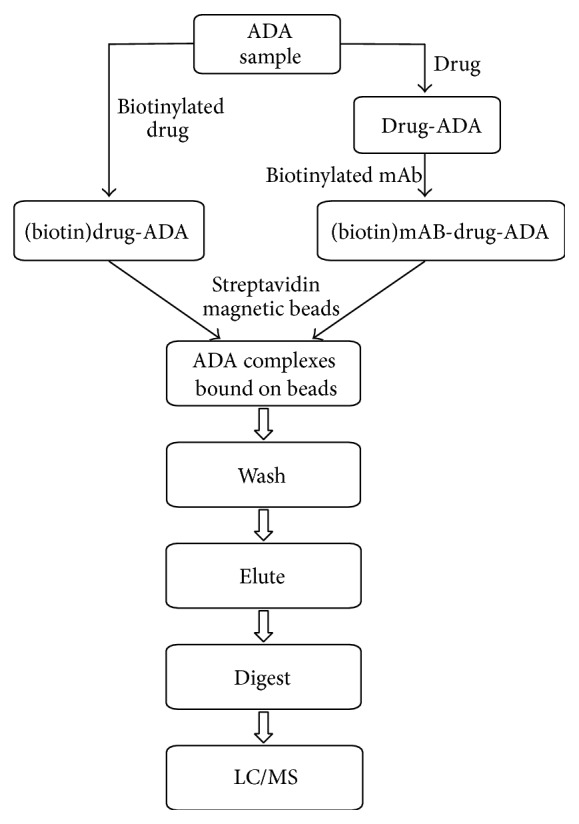
Immunocapture-LC/MS workflow chart.

After suitable surrogate peptide candidates were identified, immunocapture was performed, and sensitivity and matrix interference were assessed for each MRM pair. MRMs with the best sensitivity, specificity, and selectivity were selected. Two different immunocapture approaches were used for immunopurification. One was to capture ADAs with biotinylated Protein Z, while the other was to capture drug-ADA complexes with a biotinylated mouse mAb. Each has its own advantages and shortfalls and is discussed below in detail. The workflow of the immunocapture-LC/MS assay is depictured in Figure 1.

### 3.2. Drug as Capture Reagent

Using drug as capture reagent is straightforward, similar to traditional drug-bridging ECL assays [50]. After spiking to human plasma samples, biotinylated Protein Z bound to ADA to form (biotinylated)drug-ADA complexes. Upon adding streptavidin magnetic beads, the (biotinylated)drug-ADA complexes were immobilized on the beads and then separated from matrix using a magnet. After washing, ADAs were eluted from the beads and subjected to trypsin digestion followed by LC/MS analysis. The basis of this approach was that biotinylated Protein Z was able to capture ADAs of different isotypes/subclasses present in the samples and the ADAs can be detected by LC/MS as long as the (biotinylated)drug-ADA complexes survived the washing steps. No special reagents were required.

The primary goal of the immunocapture was to isolate ADA from the plasma matrix. The efficiency of the immunopurification should be assessed using human ADA positive controls. Because there were no human ADA standards available, commercial human Igs were used as surrogates. Although different in their variable regions, the surrogate human Igs have the same constant regions as human ADAs and thus produce the unique peptides of human ADA isotype/subclass upon trypsin digestion which can then be detected and quantitated by LC/MS. Unlike human ADA, however, the surrogate Igs would not bind to biotinylated Protein Z to form (biotinylated)drug-Ig complexes. Therefore, instead of directly spiked into blank human plasma, the surrogate Igs were spiked into the magnetic beads eluent of blank human plasma after the immunocapture steps. The spiked and unspiked magnetic bead eluent were then digested and assayed by LC/MS. Only the MRM pairs that had the best sensitivity with minimal matrix interferences were chosen for immunocapture-LC/MS assay.  Table 1 lists the final MRM pairs of each unique peptide. The MRM pairs were all y-ions with *m*/*z* greater than respective parent *m*/*z*. This was not surprising as the y-ions contained a basic amino acid (either arginine or lysine) at the N-terminal and thus showed better response in positive MS than b-ions. In addition, interference was reduced significantly when *m*/*z* values of fragment ions were greater than the doubly or triply charged parent peptide *m*/*z* values.

The final MRM pairs were grouped into two types: quantitation and confirmation. The quantitation MRMs were the most sensitive and used for isotyping and quantitation. The confirmation MRMs were less sensitive and results from the confirmation peptides are expected to be similar to those from quantitation peptides. In case there is a large discrepancy between the quantitation and confirmation peptide results, investigation on the assay may be needed. The AA sequence length was 6–12 for the quantitation peptides and 5–16 for the confirmation peptide. The quantitation peptides for IgG1, IgG2, IgG3, and IgG4 were GPSVFPLAPSSK, GLPAPIEK, WYVDGVEVHNAK, and GLPSSIEK, respectively. In addition, peptides ALPAPIEK and VVSVLTVLHQDWLNGK were also sensitive and found in IgG1/IgG3 and IgG1/IgG3/IgG4, respectively. These 2 peptides were not unique to any one single Ig isotype/subclass and were included as confirmation peptides. Likewise, quantitation peptides AEWEQK and GQPLSPEK and confirmation peptides LEVTR and VSVFVPPR were identified for IgE and IgM, respectively.

Both IgA1 and IgA2 shared the same peptide, YLTWASR, which was much more sensitive than any other unique peptides and thus used for quantitating the total of IgA1 and IgA2. In addition, peptide TFTC[CAM]TAAYPESK was unique to IgA1. However, TFTC[CAM]TAAYPESK was much less sensitive (2500 ng/mL limit of detection human plasma after immunocapture) and thus not very useful for low level ADA detection. For both IgA1 and IgA2, VAAEDWK was also used as a confirmation peptide.

**Figure 2:**
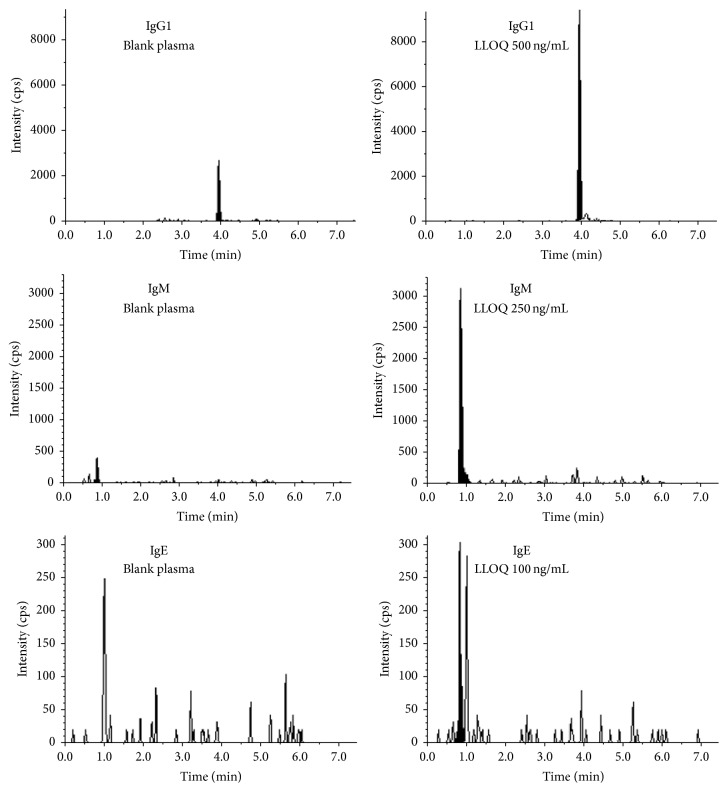
LC/MS chromatograms of unique peptides of IgG1 (top), IgM (middle), and IgE (bottom) from blank human plasma (left) and LLOQ samples (right) after immunocapture when using drug as ADA capture reagent.

After the final MRMs were selected, LC was optimized and total runtime was shortened from 20 min to 8 min. All the selected peptide came between 0.8 and 4 min in the 8 min run. Representative LC/MS chromatograms of IgG1, IgE, and IgM from the pooled ADA-free blank human plasma and low limit of quantitation standards (see discussions later) are shown in Figure 2.

The LC/MS assay was tested with blank human plasma with and without preexisting ADA (PEA) for Protein Z. The blank plasma was obtained from 20 in-house healthy donors and had been screened for PEA with a drug-bridging ECL assay. Based on the ECL assay, plasma from 9 donors was PEA negative whereas it was PEA positive from the remaining 11 donors.

The 20 plasma samples were processed using the immunocapture procedures with biotinylated Protein Z as the capture reagent. Along with the 20 plasma samples, a set of calibration standards were prepared in beads eluent from the pooled PEA negative blank plasma. After digestion, the plasma samples and the calibration standards were assayed using the LC/MS ADA method. The results were evaluated using either a cut-point or a calibration curve.

#### 3.2.1. Cut-Point with Drug as Capture Reagent

The LC/MS responses (analyte/IS peak area ratio) of each ADA isotype/subclass from the 9 PEA negative and the 11 PEA positive human plasma samples are listed in Tables 2 and 3, respectively. Similar to traditional drug-bridging assays [10], the cut-point was set at 95% to allow a rate of 5% false positives and determined with the 9 lots of PEA negative human blank plasma. The LC/MS peak area ratios of ADA IgG1 isotope ranged from 0.0060 to 0.0637, with a mean of 0.0216 and a standard deviation (SD) of 0.0177 (Table 2). Using the standard calculation formulation for 95% cut-point, mean + 1.645 × SD, the calculated cut-point value was 0.0507 for IgG1. Compared to the cut-point, 8 of 11 PEA positive samples were also ADA (IgG1) positive by the immunocapture-LC/MS assay (Table 3). The ADA (IgG1) response in the remaining 3 samples (lots 5, 7, and 8) ranged from 0.0150 to 0.0239, which was below the cut-point.

Likewise, cut-points were calculated for all other ADA isotypes/subclasses (Table 2). Based on the cut-points, there were one ADA positive plasma lot each for IgG2 and IgM and 2 for IgG3 while none for IgG4 and IgE by the immunocapture-LC/MS assay. In contrast, all these 11 samples were ADA positive for IgA1 and/or IgA2. As discussed below, except for IgG1, the LC/MS responses of all other isotypes/subclasses were below limit of quantitation (BLQ) and, therefore, their contributions to the overall ADA amounts were negligible.

#### 3.2.2. Calibration Curve with Drug as Capture Reagent

ADA levels in the 11 PEA positive samples were semiquantitatively determined using a calibration curve. A series of matrix calibration standards ranged from 0.05 to 10 *μ*g/mL were prepared in the magnetic bead eluent of pooled blank human plasma by spiking neat Ig isotype standard solutions. The matrix calibration standards were then digested and assayed by LC/MS. The calibration curve for each Ig isotype/subclass was constructed using LC/MS peak area ratios of peptide versus respective stable isotope labeled IS. Linear regression with 1/*x* ^2^ weighting was used. The ADA concentration in the plasma samples was back-calculated using the calibration curve.

During the immunocapture process, the (biotinylated)drug-ADA complexes were separated from the plasma by a magnet. However, as endogenous plasma Igs were at much higher levels [44], a small amount still remained on the beads even after the washing. The endogenous Igs surviving the washing step were carried on in subsequent elution and digestion and eventually detected as background peaks in the LC/MS assay. The endogenous interference peaks of IgG1 and IgM and IgE were clearly seen in the chromatograms of the blank plasma sample (Figure 2). The endogenous interference peak intensity seemed to follow the order of Ig abundance in human plasma [44]. IgG1 had the highest interference peak while IgE had the lowest. Similar to traditional LC/MS assay, the low limit of quantitation (LLOQ) of the calibration curve was defined such that LC/MS response at LLOQ was equal to or greater than 4x matrix background response. As shown in Figure 2, the LLOQ peaks were much higher than the corresponding matrix interference peaks and the peak areas were accurately measured. The LLOQ determined for each isotype/subclass ranged from 0.1 to 0.5 *μ*g/mL. A lower LLOQ corresponded to a higher assay sensitivity. Among the ADA isotypes/subclasses, IgG4, IgE, and [IgA1 + IgA2] had the highest assay sensitivity with LLOQ of 0.1 *μ*g/mL.

The higher limit of quantitation (HLOQ) of the calibration curve range depended on the beads and capture reagent capacity. However, as the calibration standards were prepared after immunocapture, the beads and capture reagent capacity could not be readily assessed. Based on our experiences with immunocapture using similar experimental settings, the HLOQ was arbitrarily set at 10 *μ*g/mL for all isotypes/subclasses, which should be well within the capacity of the assay.

**Figure 3:**
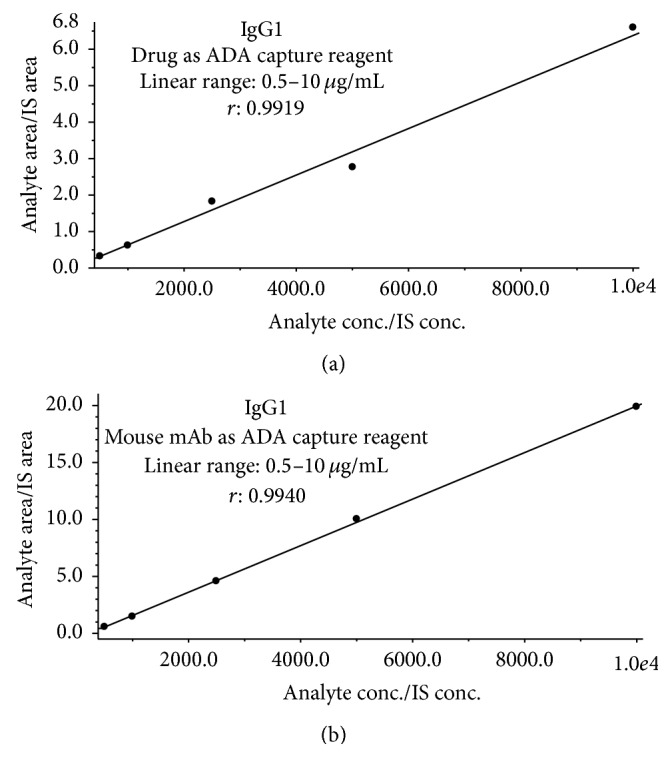
Calibration curves of IgG1 in human plasma eluent after immunocapture when using either drug (a) or mouse mAb as ADA capture reagent (b).

The calibration linear range was defined from LLOQ to HLOQ. The curve linear regression correlation coefficients (*r*) were all >0.9910 except for peptide WYVDGVEVHNAK (IgG3, which was 0.9858). The calibration linear ranges and correlation coefficients (*r*) are listed in Table 4. Representative calibration curve of IgG1 in human plasma eluent after immunocapture is shown in Figure 3.

Using the calibration curve, only 2 out of the 11 PEA positive samples had ADA levels (for IgG1 only) above the LLOQ 0.5 *μ*g/mL. Lots 3 and 11 had ADA IgG1 level of 0.660 and 0.680 *μ*g/mL, respectively. The ADA IgG1 levels in the remaining 9 samples were BLQ. It is expected that ADA IgG1 levels in some of the 9 samples could be quantitated if the LC/MS assay sensitivity was further improved. Obviously, in order to increase the assay LC/MS specificity, one has to further eliminate endogenous Igs to minimize the background response. This effort is currently ongoing in our lab. In all 11 samples, the levels of ADAs of other Ig isotypes/subclasses were BLQ. This was consistent with the fact that IgG1 is the most dominant antibody in human plasma [44].

The biggest caveat of the semiquantitation approach was that the calibration standards did not go through the immunocapture process whereas the study samples did. The calibration thus did not take immunocapture recovery into account. The recovery could be estimated using well characterized polyclonal human ADA positive controls, which were not available for Protein Z. Based on our experiences with immunocapture in similar experimental setting and those reported in literature [38], immunocapture recovery varied from project to project but usually falls within a 30–50% range. If this also held true for Protein Z, the measured ADA levels in the PEA plasma would be around 30–50% of actual concentrations.

Different from ECL assays, the ADA levels measured by the immunocapture-LC/MS represent absolute amounts. This allows one to compare ADA isotype levels between samples, studies, and different biotherapeutics. Database of such information could be gradually built and provide valuable insight to better understand immunogenicity and immunology of biotherapeutics.

As with traditional ECL drug-bridging assays, the immunocapture-LC/MS method could be hampered by drug tolerance issues when drug is present [51]. As a consequence, the assay sensitivity can be severely compromised. This limitation may be overcome by using acid dissociation to break up the drug-ADA complex and release ADA [37]. Biotinylated drug is then added to the samples so that the biotinylated drug competes with the existing drug in forming (biotinylated)drug-ADA complexes. If the amount of the biotinylated drug is much more than that of existing drug which is usually determined with a PK assay, the drug interference is greatly reduced and assay sensitivity is improved. In ECL drug-bridging assays, one binding domain of ADA has to bind to biotinylated drug while the other binds to sulfotagged drug in order to form (biotinylated)drug-ADA-(sulfotagged)drug complex and be detected. In the immunocapture-LC/MS assay, on the other hand, only one arm of ADA needs to bind to biotinylated drug and the other can still bind to the unlabeled drug. Therefore, drug interference is expected to be less in immunocapture-LC/MS assay platform.

It should be noted that if drug contains the human Ig Fc region, it may also bind to the beads via nonspecific binding just like endogenous proteins and could contribute to interference in the LC/MS assay. On the other hand, biotinylated drug that binds to streptavidin beads will not be eluted out under the elution conditions due to very strong biotin-streptavidin interaction [52]. The binding between streptavidin and biotin has long been regarded as the strongest, noncovalent, biological interaction known, with a dissociation constant *K*_*D*_ in the order of 4 × 10^−14^ M [52]. The bond forms very rapidly and is stable in wide ranges of pH and temperature [53, 54].

It was evident that the results from the immunocapture-LC/MS assay confirmed PEA positive results in most of these samples and were in good agreement with the drug-bridging ECL assay.

### 3.3. Anti-Drug Ab as Capture Reagent

The second immunocapture approach was using a mouse anti-Protein Z mAb to capture ADA in human plasma. In this approach, all ADAs had to be first completely converted to drug-ADA complexes by adding excessive Protein Z to the samples [34]. Biotinylated mouse mAb was then added to capture the Protein Z-ADA complexes along with free Protein Z. In the presence of mAb, the drug-ADA complexes and free drug were converted to drug-ADA-mAb complexes and drug-mAb complexes, respectively. After adding streptavidin magnetic beads, the complexes were immobilized on the beads and were subsequently separated from plasma using a magnet. ADA was then eluted from the beads, digested, and assayed by LC/MS following the same procedures with drug as the capture reagent described previously.

The merit using anti-drug Ab as the capture reagent lies on that drug no longer interferes with the assay. This offers a huge advantage when drug levels in the study samples are high enough such that drug tolerance becomes a concern in other types of assays. The most important element of this approach is that the capture Ab should not compete with ADA for the drug; that is, the two should not share the same binding domain on the drug. To confirm this for the mouse mAb, the immunocapture recovery of Protein Z from ADA positive samples was assessed. Protein Z was spiked at 5 ng/mL to the pooled PEA negative and the 11 positive human plasma samples and its concentration was determined using an immunocapture-LC/MS PK assay. The PK assay was developed in our lab to support clinical studies. In the PK assay, Protein Z was captured using the mouse mAb, and the resulting drug-mAb complex was then immobilized on magnetic beads, separated from plasma, eluted out from beads, digested, and analyzed by LC/MS. A unique peptide from Protein Z was monitored by LC/MS and used to quantitate Protein Z. The immunocapture recovery was determined by comparing Protein Z concentrations in the PEA positive human plasma with the pooled PEA negative plasma. No difference in Protein Z concentration was observed between the PEA positive and PEA negative samples (data not shown), and Protein Z recovery was more than 82% with averaging 97%. It was evident that the mouse mAb was indeed able to capture Protein Z regardless of whether it is in ADA-Protein Z complexes or free form. However, one has to be cautious as human ADAs come in many different forms and some may bind to the same domain on the drug as the capture Ab. Therefore, it is recommended to run this test using ADA positive samples from the study.

Similar to the first approach using drug as capture reagent, the 11 PEA positive and 9 PEA negative blank human plasma samples were used to evaluate immunocapture using the mouse mAb as the capture reagent. Besides using a different capture reagent, the only difference between the two approaches was that in the second approach there was an additional step to convert ADA to ADA-drug complexes. To ensure a complete conversion, 6 *μ*L of 5 mg/mL Protein Z buffer solution was added to 144 *μ*L of human plasma. The amount of Protein Z added was overwhelmingly more than the PEA level (≤680 ng/mL) estimated by the first approach. The same amount of Protein Z was also added to the pooled PEA blank plasma used for preparation of calibration standards.

#### 3.3.1. Cut-Point with mAb as Capture Reagent

**Figure 4:**
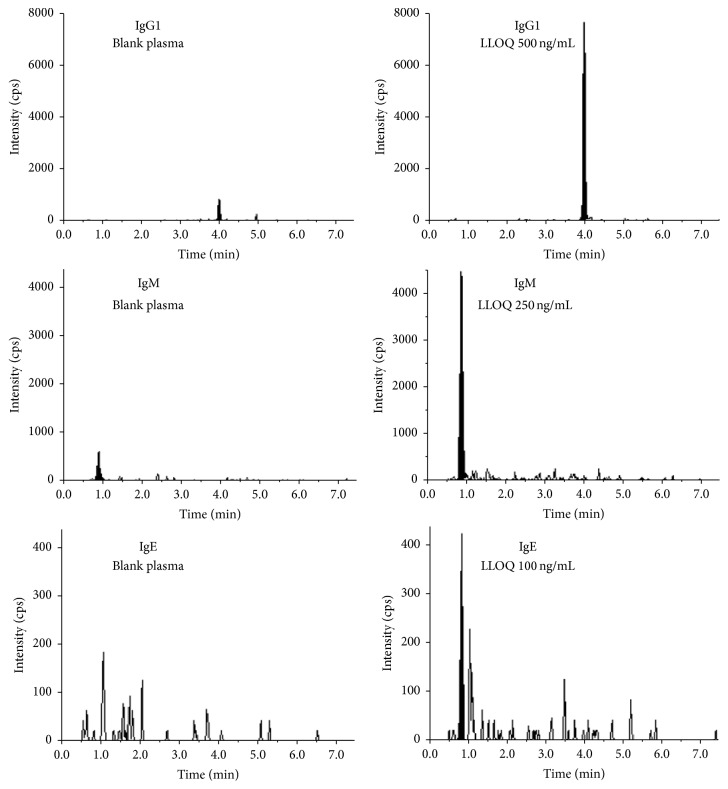
LC/MS chromatograms of unique peptides of IgG1 (top), IgM (middle), and IgE (bottom) from blank human plasma (left) and LLOQ samples (right) after immunocapture when using mouse mAb for ADA capture.

Tables 5 and 6 provide the LC/MS peak area ratio response of ADA in the PEA negative and positive samples, respectively. The LC/MS chromatograms of IgG1, IgM, and IgE unique peptides from the blank human plasma and LLOQ samples are shown in Figure 4. The LC/MS response for ADA (IgG1) from the 9 PEA negative samples ranged from 0.0210 to 0.0815, with a mean of 0.0361 and SD of 0.0194. The calculated cut-point at 95% was 0.0680 for IgG1. Using the cut-point, 7 of the 11 PEA positive samples were also ADA positive with the immunocapture-LC/MS assay. These 7 plasma lots were also ADA positive in the first approach using drug as capture reagent. Plasma lot 2 was ADA positive in the first approach but negative in the second approach. In both approaches, LC/MS response of plasma lot 2 was close to the respective cut-point, so it was not surprising to see the discrepancy between the two approaches.

Calculated cut-points for all other ADA isotypes/subclasses are listed in Table 5. Based on cut-points, lot 7 was ADA positive for IgG2, lot 8 was ADA positive for IgG3, lot 11 was ADA positive for IgM, and lots 4, 5, 6, 9, and 11 were ADA positive for IgA1 + IgA2. No positive lot was found for IgG4 or IgE. However, lot 7 for IgG2, lot 11 for IgM, and lots 6 and 11 for IgA1 + A2 were considered false positive due to the presence of interference as discussed below.

The mechanism of ADA capture using the mAb was more complicated than using drug. The ADA must be bound to the drug first, and the resulting drug-ADA complexes had to be bound to the mouse mAb and survive the immunocapture procedure in order to be detected by LC/MS. Endogenous components such as Igs that cross-reacted with the mAb could also interfere with the assay and give false positive results. Although this potential interference was already accounted for in the cut-point determination, it was further assessed for the PEA positive plasma samples without the addition of excessive Protein Z. The “−drug” plasma samples were spiked with the mAb and then processed with the immunocapture procedure followed by LC/MS analysis. The results are provided in Table 6. For IgG1, the LC/MS responses from the “−drug” samples of lots 6 (0.0731) and 11 (0.0974) were above the cut-point of 0.0680, suggesting possible interference. However, both responses were slightly above the cut-points and much less than those (0.1800 and 0.5690) from their respective “+drug” samples. Therefore, lots 6 and 11 were still considered ADA positive despite the presence of small interference. Likewise, Lot 4 was considered positive for IgA1 + A2, as the above cut-point “−drug” response (0.1380) was much less than “+drug” responses (0.2300). The remaining “−drug” positive samples, lot 7 for IgG2, lot 11 for IgM, and lots 6 and 11 for IgA1 + A2 gave similar responses as their respective “+drug” samples and thus were considered false positive. Overall, seven of the eleven PEA positive plasma samples were positive for IgG1, one was positive for IgG3, and three were positive for IgA1 + IgA2 using the mAb as ADA capture reagent.

#### 3.3.2. Calibration Curve with mAb as Capture Reagent

Calibration curves were established for each ADA isotype in the same way as in the first approach. The calibration linear ranges and curve regression correlation coefficients are provided in Table 4. Calibration curve of IgG1 is shown in Figure 3. HLOQ was also set at 10 *μ*g/mL for all isotypes/subclasses. The correlation coefficient (*r*) was >0.98 in all cases. The LLOQ, calibration ranges, and *r* were all similar to those from the first approach using drug as capture reagent.

Using the calibration curve, ADA (IgG1) level in lot 3 and lot 11 plasma was determined to be 0.570 and 1.25 *μ*g/mL, respectively. These two plasma samples were also the only ones with ADA (IgG1) level above LLOQ in the first immunocapture approach. The ADA (IgG1) level from the first approach was 13.6% and −45.6%, respectively, compared with the second approach. Given the two totally different immunocapture approaches and the limited sample size, the two sets of semiquantitative data were considered in good agreement with each other.

Besides IgG1, ADA levels for other ADA isotypes/classes were all BLQ in these 11 PEA positive samples. This was consistent with the first approach.

It should be noted that the anti-drug Ab capture approach may not be used if the biotherapeutic proteins contain constant human Fc regions. Unlike using drug as capture reagent, anti-drug Ab captures both free drug and drug-ADA complexes and during the ADA elution step drug is also eluted out from magnetic beads and thus interferes with LC/MS detection. For instance, Humira (adalimumab), a TNF inhibiting anti-inflammatory drug and the first fully human monoclonal antibody drug approved by the FDA, is an IgG1 made by phage display technology with amino acid sequences only from the human germline, making it indistinguishable in structure and function from natural human IgG1 [55]. Based on in silicon digestion prediction, Humira would yield the universal peptides of human IgG1, GPSVFPLAPSSK, and thus interfere with the universal peptide ADA assay. In this case, unique peptide(s) from the drug instead of the ADA peptides might be monitored by LC/MS and the results can be qualitatively correlated to ADA, as Neubert et al. [34] reported. Another option is to use the first immunocapture approach with biotinylated drug as the ADA capture reagent.

## 4. Conclusions

We demonstrated for the first time that immunocapture-LC/MS can be used for simultaneous ADA isotyping and semiquantitation in human plasma. Either biotinylated drug or biotinylated anti-drug Ab could be used as the immunocapture reagent, each with its own merits and shortfalls. Biotinylated drug can readily capture ADA but drug interference could be an issue if drug levels in the samples are high. On the other hand, immunocapture using an anti-drug Ab eliminates drug interference, providing that the Ab is able to capture drug-ADA complex in addition to free drug. With this method, unique peptides from each ADA isotype/subclass were identified and monitored by LC/MS. ADA isotyping was performed by the detection of isotype-unique peptides. Absolute ADA amount was determined semiquantitatively using surrogate calibration standards. Similar to traditional drug-bridging ELISA assay, cut-points at 95% were established. The assay was used for screening, isotyping, and semiquantitating preexisting ADAs in human plasma. It could be also used as a confirmatory assay. Endogenous Ig interferences need to be reduced in order to improve the assay sensitivity and specificity, and human positive ADA controls will be needed for more accurate ADA quantitation.

Owing to LC/MS's advantages such as high specificity, selectivity and reproducibility, wide dynamic range, and multiplexing capability, it is expected that, with further improvements, immunocapture-LC/MS will become an invaluable tool in immunogenicity assessment. It can be easily implemented in bioanalytical lab settings for routine ADA isotyping and semiquantitation. As ADA levels measured by immunocapture-LC/MS represent absolute amounts, one can compare ADA isotype levels between samples, studies, and different biotherapeutics, providing that consistency in positive controls is achieved to determine recovery. Database of such information could be gradually built and provide valuable insight to better understand immunogenicity and immunology of biotherapeutics.

## Figures and Tables

**Table 1:** List of unique peptides and MRMs for ADA isotopes/subclasses used in the immunocapture-LC/MS assay.

Isotype/subclass	Unique peptide sequence	MRM pairs
*Quantitation peptide*		
IgG1	GPSVFPLAPSSK	593.83→699.40
IgG2	GLPAPIEK	412.75→654.38
IgG3	WYVDGVEVHNAK	708.85→698.48
IgG4	GLPSSIEK	415.73→660.36
IgE	AEWEQK	395.69→590.29
IgM	GQPLSPEK	428.23→670.38
IgA1, IgA2	YLTWASR	448.73→620.32

*Confirmation peptide*		
IgG1, IgG3	ALPAPIEK	419.76→654.38
IgG1, IgG3, IgG4	VVSVLTVLHQDWLNGK	603.34→1110.57
IgE	LEVTR	309.18→375.24
IgM	VSVFVPPR	450.77→615.36
IgA1, IgA2	VAAEDWK	409.71→648.30
IgA1	TFTC[CAM]TAAYPESK	688.31→765.38

**Table 2:** LC/MS peak area ratio response and cut-points of ADA isotopes/subclasses in PEA negative human plasma with drug as ADA capture reagent.

Plasma lot #	IgG1	IgG2	IgG3	IG4	IgE	IgM	IgA1 + IgA2
1	0.0277	0.0099	—	0.0107	—	0.1130	0.0041
2	0.0107	0.0008	—	0.0019	—	—	0.0059
3	0.0263	—	—	0.0012	0.0150	0.0084	0.0078
4	0.0093	0.0023	—	0.0053	—	0.0072	0.0057
5	0.0249	0.0244	0.0210	0.0099	0.0110	0.0474	0.0136
6	0.0060	0.0044	0.0110	0.0035	—	0.0326	—
7	0.0129	—	—	0.0037	0.0160	0.0159	0.0062
8	0.0637	0.0205	0.0570	0.0353	0.0140	0.0066	0.0174
9	0.0127	0.0008	—	0.0111	—	0.0035	0.0075

Mean	0.0216	0.0070	0.0099	0.0092	0.0061	0.0261	0.0086
SD	0.0177	0.0093	0.0192	0.0105	0.0074	0.0361	0.0043

Cut-point (95%)	0.0507	0.0224	0.0416	0.0265	0.0183	0.0854	0.0156

—: no LC/MS response was detected.

**Table 3:** LC/MS peak area ratio responses and calculated ADA isotope/subclass levels in PEA positive human plasma with drug as ADA capture reagent (numbers in bold italic are above respective cut-points).

Lot #	IgG1	IgG1 conc. (*µ*g/mL)	IgG2	IgG3	IG4	IgE	IgM	IgA1 + IgA2
1	***0.0874***	—	**0.0033**	***0.0476***	0.0096	—	0.0499	***0.0441***
2	***0.0838***	—	***0.0452***	***0.0762***	0.0070	—	0.0421	***0.0422***
3	***0.4200***	0.660	0.0061	0.0263	0.0088	—	0.0111	***0.0847***
4	***0.1970***	—	0.0047	—	0.0020	0.0113	0.0068	***0.0497***
5	0.0239	—	0.0044	0.0119	0.0020	—	0.0007	***0.0480***
6	***0.0827***	—	0.0141	0.0205	0.0192	0.0054	0.0316	***0.0771***
7	0.0188	—	0.0041	—	—	—	0.0127	***0.0239***
8	0.0150	—	0.0109	0.0315	0.0017	0.0119	0.0027	***0.0712***
9	***0.0788***	—	0.0009	—	0.0009	0.0150	0.0099	***0.0727***
10	***0.0641***	—	0.0076	0.0192	—	0.0155	0.0314	***0.0424***
11	***0.4330***	0.680	0.0041	0.0303	0.0031	—	***0.1010***	***0.0652***

Cut point (95%)	0.0507	—	0.0224	0.0416	0.0265	0.0183	0.0854	0.0160

—: below the limit of quantitation for IgG1 concentration or no peak was detected for other isotypes/subclasses.

**Table 4:** Calibration curve ranges and regression coefficients (*r*) of ADA isotype/subclasses using drug or mouse mAb as ADA capture reagent.

Isotype/subclass	Unique peptide	Calibration curve parameters			
		Drug capture		mAb capture	
		Range (*µ*g/mL)	*r*	Range (*µ*g/mL)	*r*
IgG1	GPSVFPLAPSSK	0.5–10	0.9919	0.5–10	0.9940
IgG2	GLPAPIEK	0.25–10	0.9939	0.1–10	0.9964
IgG3	WYVDGVEVHNAK	0.25–10	0.9858	0.25–10	0.9952
IgG4	GLPSSIEK	0.1–10	0.9919	0.25–10	0.9909
IgE	AEWEQK	0.1–10	0.9947	0.1–10	0.9976
IgM	GQPLSPEK	0.25–10	0.9988	0.25–10	0.9909
IgA1 + IgA2	YLTWASR	0.1–10	0.9974	0.1–10	0.9966

**Table 5:** LC/MS peak area ratio response and cut-points of ADA isotopes/subclasses in PEA negative human plasma with mouse mAb as ADA capture reagent.

Lot #	IgG1	IgG2	IgG3	IG4	IgE	IgM	IgA1 + IgA2
1	0.0495	0.0107	—	0.1110	—	0.0422	0.0224
2	0.0340	0.0054	—	0.0357	0.0152	0.0702	0.0223
3	0.0400	0.0035	—	0.0548	—	0.0277	0.0538
4	0.0243	0.0084	—	0.0259	—	0.0358	0.0264
5	0.0265	0.0108	—	0.0677	—	0.0593	0.0237
6	0.0210	0.0511	—	0.0868	—	0.0341	0.0688
7	0.0815	0.0384	—	0.0009	—	0.0622	0.0087
8	0.0231	0.0294	—	0.0866	—	0.0029	0.0274
9	0.0253	0.0027	—	0.0719	—	0.0015	0.0094

Mean	0.0361	0.0178	—	0.0601	0.0017	0.0373	0.0292
SD	0.0194	0.0175	—	0.0345	0.0051	0.0245	0.0197

Cut-point (95%)	0.0680	0.0466	0.0000	0.1168	0.0100	0.0775	0.0617

—: no LC/MS response was detected.

**Table 6:** LC/MS peak area ratio responses and calculated ADA isotope/subclass levels in PEA positive human plasma with mouse Ab as ADA capture reagent (numbers in bold italic are above respective cut-points). Plasma samples were spiked with addition of excessive drug (+drug) or without (−drug) addition of excessive drug.

Lot #	IgG1		IgG2		IgG3		IgG4		IgE		IgM		IgA	
	−drug	+drug	−drug	+drug	−drug	+drug	−drug	+drug	−drug	+drug	−drug	+drug	−drug	+drug
1	0.0369	***0.1860***	0.0077	0.0106	—	—	0.0668	0.0823	—	—	0.0159	0.0094	0.0194	0.0411
2	0.0145	0.0563	0.0072	0.0066	—	—	0.0584	0.0458	—	—	0.0316	0.0340	0.0033	0.0113
3	0.0354	***0.2330***	0.0074	—	—	—	0.0634	0.0594	—	—	0.0268	0.0098	0.0405	0.0483
4	0.0212	***0.0715***	0.0240	0.0314	—	—	0.0484	0.0369	—	—	0.0045	0.0190	***0.1380***	***0.2300***
5	0.0350	0.0381	—	0.0059	—	—	0.0182	0.0150	—	—	0.0053	0.0118	0.0325	***0.0692***
6	***0.0731***	***0.1800***	0.0122	0.0237	—	—	0.0325	0.0544	—	—	0.0575	0.0310	***0.1810***	***0.1900***
7	0.0231	0.0343	***0.0469***	***0.0739***	—	—	0.0685	0.0768	—	—	0.0234	0.0321	0.0339	0.0476
8	0.0463	0.0474	0.0086	0.0045	—	***0.0915***	0.0572	0.0528	—	—	0.0089	0.0394	0.0142	0.0329
9	0.0396	***0.1160***	0.0011	0.0026	—	—	0.0661	0.0404	—	—	0.0094	0.0125	0.0437	***0.0681***
10	0.0188	***0.1200***	0.0020	0.0039	—	—	0.0151	0.0225	—	—	0.0275	0.0329	0.0029	0.0208
11	***0.0974***	***0.5690***	0.0094	0.0075	—	—	0.0294	0.0305	—	—	***0.2570***	***0.2850***	***0.1040***	***0.1050***

Cut-point (95%)	0.0680		0.0466		0.0000		0.1168		0.0100		0.0775		0.0617	

—: no LC/MS response was detected.
